# A novel approach for lymphatic organoid embedding: eosin pre-staining and agarose pre-embedding

**DOI:** 10.1080/21688370.2025.2472091

**Published:** 2025-02-26

**Authors:** Bao-Feng Wang, Ying-Ying Wang, Yun-Lan Yi, Ping-Ping Cao

**Affiliations:** a Department of Otolaryngology-Head and Neck Surgery, Beijing Tsinghua Changgung Hospital, School of Clinical Medicine, Tsinghua University, Beijing, P.R. China; b Department of Otolaryngology-Head and Neck Surgery, Jinzhou medical University, Jinzhou, China

**Keywords:** Adenoid organoids, agarose, eosin, histology, paraffin embedding

## Abstract

Adenoid organoids, as the primary immune barrier of the airway, provide valuable models for studying lymphatic tissue function, but their histological processing remains challenging due to their fragile structure and lack of adhesion. Here, we introduce a novel approach that combines eosin pre-staining with agarose pre-embedding to enhance visibility and structural integrity during paraffin embedding. This method simplifies sectioning and improves the quality of hematoxylin and eosin (HE) and immunofluorescence (IF) staining, yielding clear and stable signals. By addressing key limitations in lymphatic organoid processing, this technique provides a reliable solution for histological and IF studies, facilitating future research on adenoid organoids.

## Advancing lymphatic organoid embedding: challenges

Organoid technology has significantly advanced *in vitro* modeling of human tissues, providing valuable insights into organ development, disease mechanisms, and therapeutic responses. Organoids are three-dimensional (3D) cell aggregates that self-organize and differentiate into functional cell types, closely mimicking native organs. Given their structural and functional resemblance to *in vivo* tissues, organoids are often referred to as “mini-organs”.^1^ Various culture systems have been established to generate and maintain these 3D structures, with suspension culture and air-liquid interface (ALI) models being widely utilized.^2–5^

Suspension culture methods prevent direct cell attachment to the culture dish and can be scaffold-based or scaffold-free. Scaffolds, such as Matrigel, mimic the natural extracellular matrix (ECM) and provide structural support, while scaffold-free techniques rely on droplet suspension, where gravity and surface tension maintain the organoid’s 3D shape.^2,3^ Alternatively, the ALI method, originally developed for skin and respiratory epithelial cells, has been successfully adapted for lymphatic organoids, including tonsil-derived models. This system allows lymphocytes to aggregate and form structures that closely resemble them *in vivo* counterparts.^4,5^

Despite these advancements, embedding lymphatic organoids for histological analysis remains a challenge. The adenoid, a critical secondary lymphatic organ in the upper airway, plays an essential role in immune defense. However, due to its floating growth characteristics *in vitro*, embedding adenoid organoids for histological sectioning presents unique technical difficulties. Traditionally, researchers have utilized eosin pre-staining or agarose/egg white pre-embedding for epithelial and tumor organoids,^6–8^ but these methods have not been adapted for lymphatic models. Tonsil organoids, another lymphatic model, have been processed via ice-sectioning, yet this technique is labor-intensive and difficult to implement in routine workflows.

## A dual-step embedding strategy for enhanced structural integrity

**Figure 1.**
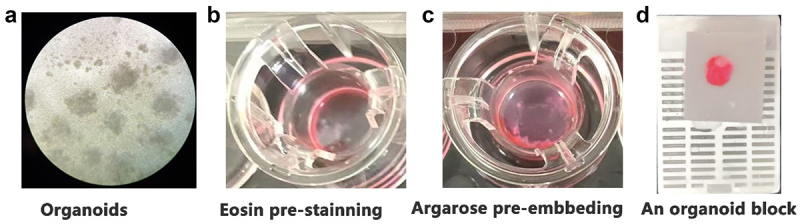
Workflow of eosin pre-staining and agarose pre-embedding for adenoid organoids.

To overcome the challenges in histological processing of ALI-cultured organoids, we propose a refined two-step embedding strategy that integrates eosin pre-staining with agarose pre-embedding (Figure 1(a–d)). Eosin pre-staining enhances organoid contrast, improving visibility and precise localization during processing, while agarose embedding provides essential mechanical support, preventing sample fragmentation during paraffin embedding and sectioning. This optimized approach preserves organoid architecture, ensuring structural integrity and facilitating high-quality histological and immunofluorescence (IF) analysis.

Preparation of a paraffin-embedded organoid block representative images illustrating the key steps in organoid processing. Organoids observed under a microscope at 40× magnification (a) Eosin pre-staining enhances contrast, captured under a normal camera at 1× magnification (b) Organoids pre-embedded in eosin-stained agarose to preserve structural integrity, imaged under a normal camera at 1× magnification (c) The final paraffin-embedded organoid block, prepared for histological processing and sectioning, captured under a normal camera at 1× magnification (d).

## Advancing immunophenotyping in lymphatic organoids

**Figure 2.**
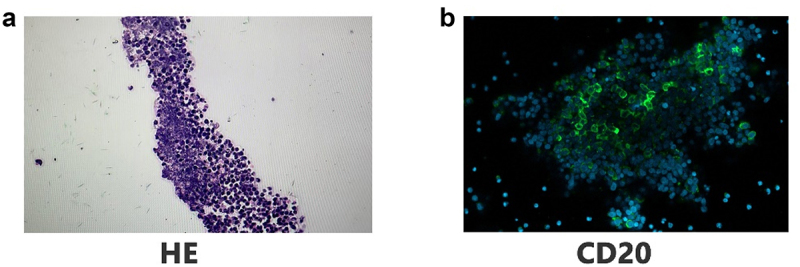
Representative HE and IF staining results, demonstrating clear structural preservation and robust marker detection.

The effectiveness of this technique was validated using adenoid organoids cultured via an ALI system. Compared to conventional methods, eosin-agarose embedding significantly improved hematoxylin and eosin (HE) staining quality (Figure 2(a)), preserving organoid architecture with minimal distortion. Additionally, IF staining of CD20, a key marker for B cells, demonstrated strong and stable signals (Figure 2(b)), reinforcing the method’s suitability for immunophenotypic analysis. The incorporation of eosin-stained agarose also enhanced contrast, enabling precise sectioning and facilitating detailed histological evaluation.

HE and IF analysis of organoid sections HE staining of organoid sections observed under a light microscope at 400× magnification, showing the overall cellular morphology (a) IF staining for CD20 (green), a B-cell marker, observed under a fluorescence microscope at 400× magnification, indicating the presence of B lymphocytes within the organoid structure. Nuclei are counterstained with 4’,6-diamidino-2-phenylindole dihydrochloride (DAPI; blue) (b).

## Beyond conventional techniques: a more accessible and reproducible alternative

Paraffin embedding remains the gold standard for histological studies, yet existing protocols often fail to maintain the integrity of ALI-cultured lymphatic organoids. While ice-sectioning offers an alternative,^5^ it requires specialized equipment and expertise, limiting its routine use. In contrast, the eosin-agarose technique provides a cost-effective, reproducible, and efficient solution that enhances sample retention and visualization, making it a more practical option for laboratories working with lymphatic organoids.

## Future directions: expanding the scope of organoid research

By refining the embedding process for lymphatic organoids, this method bridges a critical gap in organoid histology, paving the way for more robust immunological studies. Future research should explore its broader applications beyond adenoid organoids, including other non-epithelial models such as immune and tumor-derived organoids. As organoid technology continues to evolve, optimized embedding strategies will be essential for unlocking new insights into tissue biology, immune interactions, and disease pathophysiology.

## Conclusion

This dual-step approach enhances organoid contrast and stability, addressing key challenges in lymphatic organoid histology. By improving structural integrity and compatibility with standard embedding workflows, it offers a reproducible solution for histological and IF analysis. Its adaptability to other organoid models may further expand its relevance in biomedical research.
